# Antiproliferative Effect of Aaptamine on Human Chronic Myeloid Leukemia K562 Cells

**DOI:** 10.3390/ijms12117352

**Published:** 2011-10-26

**Authors:** Meihua Jin, Wennan Zhao, Yanwen Zhang, Motomasa Kobayashi, Hongquan Duan, Dexin Kong

**Affiliations:** 1 Tianjin Key Laboratory on Technologies Enabling Development of Clinical Therapeutics and Diagnostics, School of Pharmaceutical Sciences and Research Center of Basic Medical Sciences, Tianjin Medical University, Tianjin 300070, China; E-Mails: jinmeihua@tijmu.edu.cn (M.J.); wennan0719@yahoo.cn (W.Z.); zhangyanwen@tijmu.edu.cn (Y.Z.); 2 Graduate School of Pharmaceutical Sciences, Osaka University, Yamada-oka 1-6, Suita, Osaka 565-0871, Japan; E-Mail: kobayasi@phs.osaka-u.ac.jp

**Keywords:** aaptamine, K562 cells, G2/M arrest, p21

## Abstract

We previously isolated aaptamine, a benzonaphthyridine alkaloid, from marine sponge *Aaptos suberitoids*. In this study, we investigated the anti-proliferative effect of aaptamine on chronic myeloid leukemia (CML) K562 cells. Aaptamine inhibited growth of K562 with a GI50 as 10 μM, and arrested cell cycle at G2/M phase. Western blot analysis indicated that aaptamine induced p21 expression in K562 cells. Moreover, p21 promoter was activated by aaptamine treatment in p21 transfected K562 cells. Since K562 is p53 negative, aaptamine was demonstrated to be a p53-independent p21 inducer in CML cells.

## 1. Introduction

Chronic myelogenous leukemia (CML) is a myeloproliferative disease originating from a constitutively active tyrosine kinase termed Bcr-Abl, which is expressed by an oncogene resulting from a reciprocal translocation between chromosome 9 and chromosome 22 [1]. As the first molecular-targeted small molecular anticancer drug, Glivec (imatinib), an inhibitor targeting Bcr-Abl, has been used to treat CML. In the last decade, Glivec exhibited remarkable efficacy in treatment of CML patients particularly in chronic phase [2]. However, resistance in some patients has been frequently reported [3]. The resistance is mainly caused by mutations in the Abl tyrosine kinase domain, which affects binding of Glivec and its analogues [4,5]. Therefore, development of an alternative drug to Glivec for CML treatment has been expected.

Discovery of antitumor drugs from marine organisms has made great progress in recent years. Trabectedin was the first antitumor drug which was approved in European Union in 2007 [6]. Moreover, Halaven^R^ (eribulin mesylate), an analogue of halchondrin B isolated from marine sponge *Halichondria Okadai*, was approved by FDA of USA for the treatment of metastatic breast cancer on 15 November 2010.

**Figure 1:**
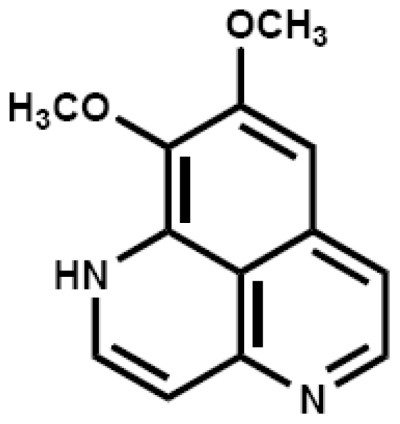
Chemical structure of aaptamine.

Aiming for discovery of new antitumor drug candidates or seed compounds from marine organisms, we have been carrying out bioassay guided isolation of compounds from marine sponges or screening of the isolated compounds with novel assay methods [7–12]. We previously isolated aaptamine (Figure 1) from the marine sponge of *Aaptos suberitoides* and reported the p21-transactivating activity on human osteosarcoma MG63 cells [7]. Whether this compound has anti-proliferative effect on CML or not remained unclear. Recently we investigated the *in vitro* activity of aaptamine on proliferation, cell cycle progression, *etc*., in K562 cells.

## 2. Results and Discussion

### 2.1. Aaptamine Inhibited the Proliferation of K562 Cells

**Figure 2:**
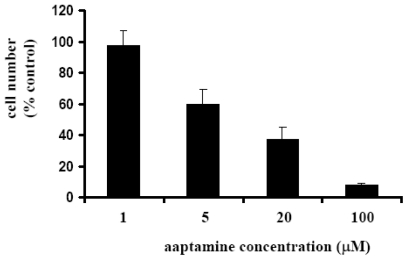
Effect of aaptamine on growth of K562 cells. K562 cells were incubated with various concentrations of aaptamine in 96-well plate at 37 °C. Two days later, WST-8 solution was added and the cells were further incubated for 3 h followed by measurement of the absorbance (450 nm) with microplate spectrophotometer. The number of remaining cells after treatment was calculated using the following formula: Cell number (% control) = 100 × (absorbance of a given sample − absorbance of Blank well)/(absorbance of Control well − absorbance of Blank well), where the Blank well contained medium only and the Control well contained cells without aaptamine.

We first examined the anti-proliferative activity of aaptamine on K562 cells by use of WST-8 assay as described by us previously [13]. After treatment with various concentrations of aaptamine for 48 h, remaining cell number was measured. As shown in Figure 2, aaptamine inhibited the growth of K562 cells dose-dependently. The GI50 (the concentration that causes 50% inhibition of the growth of cells) was calculated to be 10 μM by Graphpad Prism 4.

### 2.2. Aaptamine Arrested the Cell Cycle of K562 Cells at G2/M Phase

**Figure 3:**
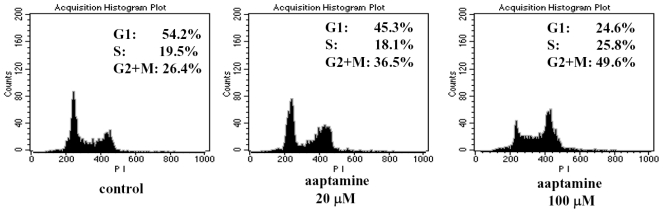
Effect of aaptamine on the cell cycle of K562 cells. K562 cells was incubated with various concentrations of aaptamine for 48 h. The collected cells were dyed with DNA-Prep Reagents Kit and analyzed by flow cytometer (λex = 493 nm, λem = 630 nm). The analysis was quantified by ModFit software.

Since cell cycle progression is required for cell growth and proliferation, we investigated the effect of aaptamine on the cell cycle progression in K562 cells. The cells were treated with 0, 20 and 100 μM of aaptamine for 48 h and the cell cycle was analyzed by flow cytometer. As shown in Figure 3, cells accumulating in G2/M phase increased while those in G1 phase decreased dose-dependently; particularly, 100 μM of aaptamine accumulates the K562 cells in G2/M phase to 49.6%, suggesting that aaptamine mainly arrests K562 cells at the G2/M phase. In addition, there was no obvious sub-G1 phase, suggesting no obvious apoptosis induced by aaptamine treatment.

### 2.3. Aaptamine Induced p21 Expression in K562 Cells

Cell cycle progression is promoted by Cdk (cyclin dependent kinase)-cyclin complexes and inhibited by cyclin-dependent kinase inhibitors (CKIs), which suppress kinase activity of Cdk-cyclin complexes. As a well known CKI, p21 is mainly controlled by diverse mechanisms in a p53-dependent manner. P53 was known to be loss-of-function mutated in over 50% of human cancer cells [14,15], as a major event of carcinogenesis. Therefore, the agents that induce an increase of p21 expression in a p53-independent manner are considered to contribute to cancer chemotherapy or prevention.

**Figure 4:**
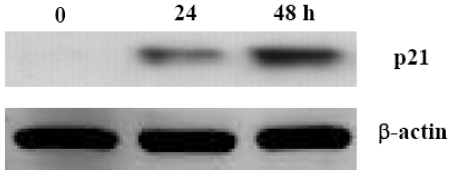
Aaptamine induced p21 expression in K562 cells. The cells were incubated with 100 μM of aaptamine for the indicated times. Cell lysates were prepared and applied to 4–20% SDS-Polyacrylamide gel electrophoresis (SDS PAGE). After being transferred to polyvinylidene difluoride (PVDF) membrane, the blots were exposed to anti-Cip1/WAF-1/p21 or anti-β-actin, and then to anti-mouse horseradish peroxidase (HRP) conjugated IgG antibody.

So we examined whether aaptamine could induce p21 expression in K562 cells, which are known to be p53 negative. K562 cells were treated with 100 μM of aaptamine and incubated for 0, 24, and 48 h, respectively. The cells were harvested for western blot analysis. As shown in Figure 4, the expression of p21 was not detectable before treatment. After treatment with aaptamine, p21 expression was induced and increased time-dependently within 48 h. 10 μM of aaptamine led to similar effect on p21 expression, but less obvious than 100 μM.

### 2.4. Aaptamine Activated p21 Promoter in K562 Cells

**Figure 5:**
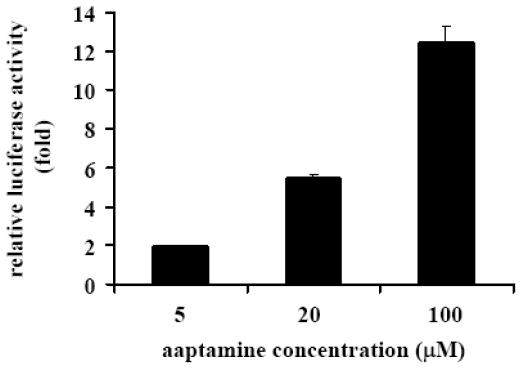
Activation of p21 promoter in K562 cells. K562 cells were transfected with the human p21 promoter-luciferase fusion plasmid (pWWP), followed by treatment with various concentrations of aaptamine for 24 h, and then analyzed by luciferase assay. Fold increase of luciferase activity by aaptamine was calculated in comparison with the luciferase activity of the untreated cells. The result represents three independent experiments.

To investigate whether the induction of p21 expression is caused by the activation of p21 promoter, the activating effect of aaptamine on p21 promoter in K562 cells transfected with a human p21 promoter-luciferase fusion plasmid (pWWP) was examined. The relative luciferase activity of the aaptamine-treated cells indicates an intensity of the activation of p21 promoter in comparison with that of the untreated cells. As shown in Figure 5, aaptamine activated p21 promoter dose-dependently and increased the luciferase activity to 12.5 fold at 100 μM. Since K562 cell is p53 negative cell, the activation of p21 promoter and the above-mentioned p21 expression induction is supposed to be p53-independent.

## 3. Experimental Section

### 3.1. Materials

WST-8 assay kit was purchased from Kishida Chemicals. DNA-Prep Reagents Kit was from Coulter Co., Ltd. Polyvinylidene difluoride (PVDF) membrane was purchased from Amersham Pharmacia Biotec UK, Ltd. Gel cassettes (4–20%) were from Daiichi Pure Chemicals Co., Ltd. Anti-Cip1/WAF-1/p21 antibody was from Milipore. Anti-β-actin was from Sigma.Anti-mouse HRP-conjugated antibody was from Nacalai Tesque Inc. PWWP, a human p21 promoter-luciferase fusion plasmid, was prepared as described by us previously [7].

### 3.2. Isolation and Identification of Aaptamine

Aaptamine was isolated from the marine sponge *Aaptos suberitoids* as described previously [8]. The structure was identified by comparison of the mass and NMR data with those reported.

### 3.3. Cell Culture

Human chronic myeloid K562 cells were routinely maintained in the RPMI medium supplemented with 10% fetal bovine serum, 100 μg/mL of kanamycin, and 0.44 mg/mL of glutamine at 37 °C in a humidified atmosphere containing 5% CO_2_.

### 3.4. WST-8 Assay to Determine K562 Cell Growth

Cell viability was determined using the WST-8 assay kit as described previously by us [11,13] with a small modification. To investigate the effect of aaptamine on the growth of K562 cells, 0.1 mL of cells (8 × 10^3^ cells/well) was seeded in 96-well plate in RPMI medium at 37 °C in a humidified atmosphere containing 5% CO_2_. Twenty four hours later, 0.5 μL of various stock solutions of aaptamine was added to achieve different final concentrations. After further incubation for 48 h at 37 °C, 10 μL of WST-8 was added to each well and the cells were further incubated at 37 °C. Three hours later, the absorbances at 450 nm and 650 nm (background) were measured with a microplate spectrophotometer. The number of viable cells remaining after the treatment was calculated using the following formula: Cell number (% control) = 100 × (absorbance of a given sample—absorbance of Blank well)/(absorbance of Control well – absorbance of Blank well), where the Blank well contained medium only and the Control well contained cells without aaptamine. The GI50 value was calculated by fitting the data points to a logistic curve using the GraphPad Prism 4 software.

### 3.5. Flow Cytometric Analysis of Cell Cycle

The suspension (2 × 10^5^ cells/2 mL/well) of K562 cells was placed in an 8-well plate and incubated for 24 h at 37 °C under a 5% CO_2_ atmosphere. Various concentrations of aaptamine (0, 20, 100 μM) were added and further incubated for 48 h. Then the cells were harvested and washed twice with cold PBS. The cells were then dyed with DNA-Prep Reagents Kit for 20 min. After centrifugation at 1000 × g, the supernatant was removed. 500 μL of PBS was added to the cell pellet, and the cell suspension was filtered with a 40 μm nylon mesh filter for cell cycle analysis. The analysis was carried out by flow cytometer (FACS Calibur, Beckton Dickinson, λex = 493 nm, λem = 630 nm) and quantified by ModFit Software.

### 3.6. Western Blot Analysis

The cell suspension (1 × 10^6^ cells/8 mL) of K562 cells was incubated with 100 μM of aaptamine for the indicated times under a 5% CO_2_ atmosphere at 37 °C. The cells were harvested and treated with lysis buffer (50 mM Tris-HCl, pH 7.2; 1% NP-40; 0.25% sodium deoxycholate; 150 mM NaCl; 1 mM EDTA; 1 mM PMSF; 1% proteinase inhibitor cocktail) to furnish a cell lysate. Protein assay was carried out by Bio-Rad protein assay kit. After boiling at 95 °C for 5 min in the sample buffer (0.125 M Tris-HCl, pH 6.8; 10% 2-mercaptoethanol; 4% SDS; 10% sucrose; 5% bromophenol blue), the equal amounts of protein were subjected to SDS-polyacrylamide gel electrophoresis (SDS-PAGE) and then transferred to PVDF membrane. The membrane was blocked with 5% milk TBS (Tween PBS), exposed to anti-Cip1/WAF-1/p21 or anti-β-actin and then to the anti-mouse HRP-conjugated antibody. The bound antibody was finally visualized by Enhanced Chemiluminescence (ECL) system.

### 3.7. Transfection of K562 Cells and Luciferase Assay

K562 cells were transfected with a human wild-type p21 promoter luciferase fusion plasmid pWWP by DEAE-Dextran (CellPhect Transfection Kit, Amersham Pharmacia Biotech, Uppsala, Sweden) as described by us previously [7]. The cells were incubated at a density of 5 × 10^4^ cells/mL in a 12-well plate for 24 h. Then transfection was performed by adding pWWP and incubating for 15 min. The transfected cells were further incubated for 24 h followed by treatment with various concentrations of aaptamine for another 24 h. Finally, the cells were collected for luciferase assay using the luciferase assay system (E1501, Promega, Madison, USA) as we reported [7], and the luminescence was measured by using MICRO LUMAT Plus LB96V luminometer and WING LOW software. The activation of p21 promoter was evaluated by the relative light intensity compared with that of the control (cells treated with DMSO only).

## 4. Conclusions

Aaptamine, an alkaloid isolated from the marine sponge of *Aaptos suberitoides*, inhibited growth of K562 cells, arrested cell cycle at G2/M phase, and transactivated p21 promoter in a p53-independent manner. We noted that aaptamine was recently reported to inhibit proteasome, while this activity was shown to be unrelated to the antiproliferative effect [16]. To our knowledge, our study is the first report about the antiproliferative activity of aaptamine on CML cells. To further demonstrate the antiproliferative effect of aaptamine on CML cells, activities on other CML cell lines such as KYO-1 and KCL-22 need to be tested. We also determined the antiproliferative activity of aaptamine on some other cancer cell lines such as A549 (lung cancer) and PC-3 (prostate cancer) with the GI_50_ as 7 and 10 μM, respectively, suggesting that the antiproliferative effect of aaptamine is not specific to CML cells. While aaptamine might not become an anticancer drug itself, it might be used as a chemical tool to investigate the p21-related biology.
